# Biochemical and histological alterations induced by the smoke of allethrin based mosquito coil on mice model

**DOI:** 10.1186/s12907-017-0057-9

**Published:** 2017-08-30

**Authors:** M. Abdulla Al-Mamun, M. Ataur Rahman, M. Habibur Rahman, K.M.F. Hoque, Z. Ferdousi, Mohammad Nurul Matin, M. Abu Reza

**Affiliations:** 1 Department of Biotechnology, The University of Tokyo, Bunkyo-ku, Tokyo, Japan; 2 Protein Science Lab, Department of Genetic Engineering and Biotechnology, University of Rajshahi, Rajshahi, 6205 Bangladesh

**Keywords:** Allethrin, Apoptosis, Histology, Liver, Lung, Mice and mosquito coil

## Abstract

**Background:**

Mosquito coil (MC) emits insecticide upon burning which provides limited protection against lethal mosquito borne diseases. However, apart from killing the insect, toxicities associated with the inhalation of these insecticides poses severe health hazards. However, the use of MC is increasing day by day in third world countries in particular but, yet to receive enough attention of both policy maker and general public. The current study was aimed to assess the MC smoke induced damage of pulmonary and hepatic tissues along with observing the alterations of several blood biochemical parameters in mice model.

**Methods:**

A total of twenty four Swiss albino mice were allowed to inhale the smoke of allethrin based MC at different duration per day for 120 days. By the end of treatment period, blood sample was drawn from each mouse and blood biochemical parameters including alanine transaminase (ALT), aspartate transaminase (AST), blood urea nitrogen(BUN), serum total protein, cholesterol, low density lipoprotein (LDL) and triglyceride (TG) were analyzed. Intact lung and liver were collected for histological analysis using standard protocol.

**Results:**

Biochemical study indicates elevated activity of two hepatic enzymes: ALT (89%), AST (85%), in comparison with the respective control. Increased level of some parameters of lipid profile including cholesterol (36%), LDL (48%) and triglyceride (30%) in smoke inhaled mice is the new finding of this study. On the contrary, the activity of serum total protein and BUN was decreased by 20% and 24%, respectively in inhaled mice. Pulmonary tissue of treated mice shows severe forms of emphysema and hyperplasia, especially in the peripheral region of lung, which is the hallmark of chronic obstructive pulmonary disease (COPD). Histological study of hepatic tissue shows apoptosis mediated damage of hepatocytes along with severe form of necrosis. Infiltration of Inflammatory cells was also observed in both of the organs.

**Conclusion:**

Results from the present studies suggest that chronic exposure of allethrin based MC is responsible factor for severe health complications such as COPD due to the alterations of the key biochemical parameters of blood and histo-organization of lung and liver.

## Background

Mosquito coil is the slow-burning structure made mainly of insecticides along with inert materials such as wood floor, coconut shell powder, starch etc. Upon burning, MC emits smokes, containing single or multiple insecticides, which creates a defending environment and protects the subject from several mosquito borne lethal diseases including malaria, filaria and dengue. Pyrethroids and Pyrethrins are the main active ingredients of MC. Among the several types of pyrethroid, allethrin especially d-transallethrin (belong to the type I pyrethroids) is being used in almost all brands of mosquito coil in South Asian territory. Allethrin is a type of neurotoxin, acting both on the peripheral and central nervous systems by modifying the kinetics of voltage-sensitive sodium channel, resulting in increasing of sodium permeability across the channel and paralysis of insect’s organs [1]. On the other hand pyrethroids have well been reported to induce oxidative stress and alter antioxidant level in different organ systems of rodent animal [2, 3].

MC smoke is considered as a potent air pollutant in indoor environment. One MC, upon burning, produces such amount of particulate matter equivalent to that of 75–137 cigarettes [4]. Along with insecticides, MC smoke also contains sub-micrometer particles, volatile, semi-volatile organic compounds and gaseous pollutants such as polyaromatic hydrocarbons [5, 6]. After using the MC in a room, it was observed that the maximum concentration of allethrin (0.0120 ppm) prevails till 30–35 min followed by a gradual decline in 6 h [7]. During overnight burning, subjects at close proximity are exposed to sub-micrometer particles, metal fumes, free radicals and vapors from smoke which ultimately reaches the alveolar region of lung and leading to irritation of the upper respiratory tract [6]. Chronic inhalation of MC smoke has been reported to correlate with asthma and persistent wheeze in human [8] along with focal declination and metaplasia in tracheal epithelium in rodents [5, 9]. Prolong exposure and subsequent entry of allethrin along with other MC derived particulate matters into red blood cells lead to the alteration of blood biochemical parameters including hemoglobin percentages [10, 11]. Exposure of pyrethrin based MC smoke has also been reported to considerable increase in white blood cell (WBC), especially basophil and lymphocyte in rat [12]. This noxious synthetic chemical is proven to be responsible for cellular, tissue and organs injury due to exerting its action on the membrane phospholipids, resulting membrane fluidity and tissue damage [11, 13].

The toxic smoke, emitted from MC is also reported to be responsible for inducing chromosomal aberrations in pulmonary alveolar macrophages and bone marrow [14, 15]. Recently, Madhubabu et al., (2012) have shown that MC smoke induces elevated level of reactive oxygen species (ROS) as well as over-expression of stress responsive gene, p53 [16]. Oxidative stress (DNA damage mediated) induced up-regulation of p53 is considered as the critical and early event of mitochondria mediated apoptosis [17]. Therefore, it could be postulated that MC smoke would induce apoptotic mediated tissue damage in subject with prolong exposure.

Subtropical climate, Poor drainage system and dense population are triggering the mosquito pressure day by day in tropical region including Bangladesh. As a cheap and instant remedy, middle and lower income groups are regularly using MC. Thus, a huge number of people are undesirably exposing to toxic fine particles and free radicals present in the smoke of MC and are subjecting themselves to various health complications regularly [18]. Therefore, this study was aimed to analyze the patterns of damages in the histo-architecture of lung and liver tissues as well as the alteration of biochemical parameters of blood in mice model inhaled with the mosquito coil smoke.

## Methods

### Chemical and reagents

Picric acid, Ethanol, glacial acetic acid and diethyl-ether (Sigma, USA); ALT, AST and BUN kit (Human, Germany), total protein reaction reagents (Spinreact, Spain), Cholesterol, triglycerides, LDL cholesterol kit (SIEMENS Laboratories Ltd., UK) and all other chemicals used in the experiments were of international standard.

### Experimental animal, ethics statement, mosquito coil and inhalation treatment

A total of thirty female Swiss albino mice (25-30 g) were purchased from the department of Pharmacy, Jahangirnagar University, Dhaka, Bangladesh. The animals were kept in the animal house of the department of Biochemistry and Molecular Biology, University of Rajshahi, Bangladesh for acclimatization in the experimental environment.

The protocols used to carry out the current study along with handling and caring of experimental animals were reviewed, assessed and approved by the Institutional Animal, Medical Ethics, Biosafety and Biosecurity Committee (IAMEBBC) for Experimentations on Animal, Human, Microbes and Living Natural Sources (license no: 225/320-IAMEBBC/IBSc), Institute of Biological Sciences, University of Rajshahi, Bangladesh.

Mosquito coil, used in this study was purchased from local super market of Rajshahi by the trade name of Mortein power booster coil from Reckitt Benckiser, Bangladesh Ltd. The active ingredient of this brand was 0.12% *w*/w d-*tans*allethrin and rest of the composition (99.88% *w*/w) was inert materials.

Forty days old mice were randomly distributed into control and inhaled group: Control group (six mice) was allowed to receive normal air at separate chamber. The inhaled group was then divided again into four subgroups (each containing six mice): group-1, group-2, group-3 and group-4. Each of these groups was allowed to inhale the smoke of MC at the duration of ½, 1, 2 and 3 h/d, respectively for 120 days. Inhalation treatment was conducted in limited ventilated space, creating a closed door setting mimicking the flat house.

### Biochemical study

Blood sample was drawn from caudal vena cava of each mouse by direct punching of syringe (3 mm) into the vain. Collected blood sample was kept for half an hour and then centrifuged at 5000 rpm for 10 min at 4 °C to separate serum as clear supernatant and stored at −20 °C for biochemical analysis.

The activity of ALT and AST was determined by colorimetric method, described previously by Reitman and Frankel [19]. BUN level was estimated by enzymatic colorimetric method [20]. Serum total protein was measured by Biuret method, originally described by Josephson et al. [21]. Total cholesterol was estimated by enzymatic-(hydrolysis and oxidation) colorimetric reaction (CHOD-PAP- method), described originally by Allain et al. [22]. Activity of TG and LDL were determined by enzymatic colorometric reaction (GPA-PAP-Method) [23, 24].

### Histological study

Histological study was carried out using the protocol previously described by Carleton et al. [25]. In brief, at the ending of treatment period, animals from each group were anesthetized with diethyl-ether and sacrificed by cervical dislocation. Intact lung and liver tissues were surgically removed quickly and washed with 0.9% normal saline followed by fixing with Bouin’s solution for overnight. Tissues were then washed thoroughly under running tap water and subsequently passed through gradually increasing concentrations of alcohol (from 70% to 100%) for dehydration followed by embedding in paraffin. The paraffin blocks containing tissue were sectioned at 6 μm thickness by rotary microtome (SHIBUYA, optical Co LTD, Tokyo, Japan) and stained with hematoxiline and eosin solution. Finally, the slides were visualized under light microscope (optika, Italy) and photographs were taken by the camera attached to it.

### Statistical analysis

Statistical analysis for the assessment of the alterations of biochemical parameters of blood comparing with the respective control (pair comparison) was performed using student’s *t*-test method. Descriptive statistical analysis was performed to estimate mean and standard deviation. Additionally, “p for trend” test was carried out using regression method to evaluate the relationship between the effect and treatment doses as a continuous variable. Data was expressed as mean ± SD (*n* = 6). The significance was set at *P* < 0.05. All analysis was performed using ‘STATA’ software, version 12.

## Results

### Biochemical alterations of blood parameters

Toxicological effects of allethrin based MC on different biochemical parameters of blood are presented in Table 1. The blood profiles, representing liver function, show significant changes in smoke inhaled groups in comparison with their respective control. Data indicates that the activity of two key hepatic enzymes i.e. ALT and AST were increased significantly (at *P* < 0.05) by 89% and 85%, respectively in mice exposed to MC smoke for 3 h/d. On the other hand, with the same exposure, the activity of BUN and total protein was decreased by 24% and 20% respectively. Increased levels of cholesterol, LDL and triglyceride were observed in the treated mice, and the values in 3 h/day inhaled mice were higher by 36%, 48% and 30% than the control, respectively. Trends for *p* value indicates that when the treatments duration were increased, the activity of ALT, AST, cholesterol, LDL and triglyceride were increased as well as BUN and total protein concentration were decreased as a continuous variable. Taken together, the biochemical parameters were altered by the inhalation with the mosquito coil smoke in a dose-dependent manner.

**Table 1:** Inhalation effects of the mosquito coil smoke on biochemical parameters of blood

Parameters	Control	Inhalation treatment				Trend for *p* value
		Group 1 (1/2 h)	Group 2 (1 h)	Group 3 (2 h)	Group 4 (3 h)	
ALT (U/L)	10.20 ± 1.32	11.03 ± 0.92	12.66 ± 2.44*	14.83 ± 2.86*	19.30 ± 2.00*	+0.001
AST (U/L)	41.10 ± 3.26	47.83 ± 4.18*	56.20 ± 4.10*	62.62 ± 5.8*	76. 96 ± 7.46*	+0.001
BUN (mg/dl)	9.66 ± 0.68	8.66 ± 0.75	8.46 ± 0.66	7.93 ± 0.41*	7.33 ± 0.51*	−0.001
Total protein (g/dl)	5.40 ± 0.32	5.13 ± 0.24	5.2 ± 0.23	4.8 ± 0.22*	4.32 ± 0.19*	−0.001
Cholesterol (mg/dl)	99.0 ± 4.68	105.3 ± 6.02	116.6 ± 4.93*	128.4 ± 9.45*	135.6 ± 8.11*	+0.001
LDL (mg/dl)	64.66 ± 3.51	69.43 ± 7.78	75.20 ± 5.0*	88.0 ± 6.88*	95.3 ± 8.16*	+0.001
Triglyceride(mg/dl)	86.8 ± 2.77	93.0 ± 5.36	96.66 ± 4.68*	100.3 ± 6.08*	112.58 ± 7.3*	+0.001

Data represent as averages ± SD (*n* = 6), significance was set at *P < 0.05 with respect to the control group

### Histological changes in pulmonary tissues

**Fig. 1:**
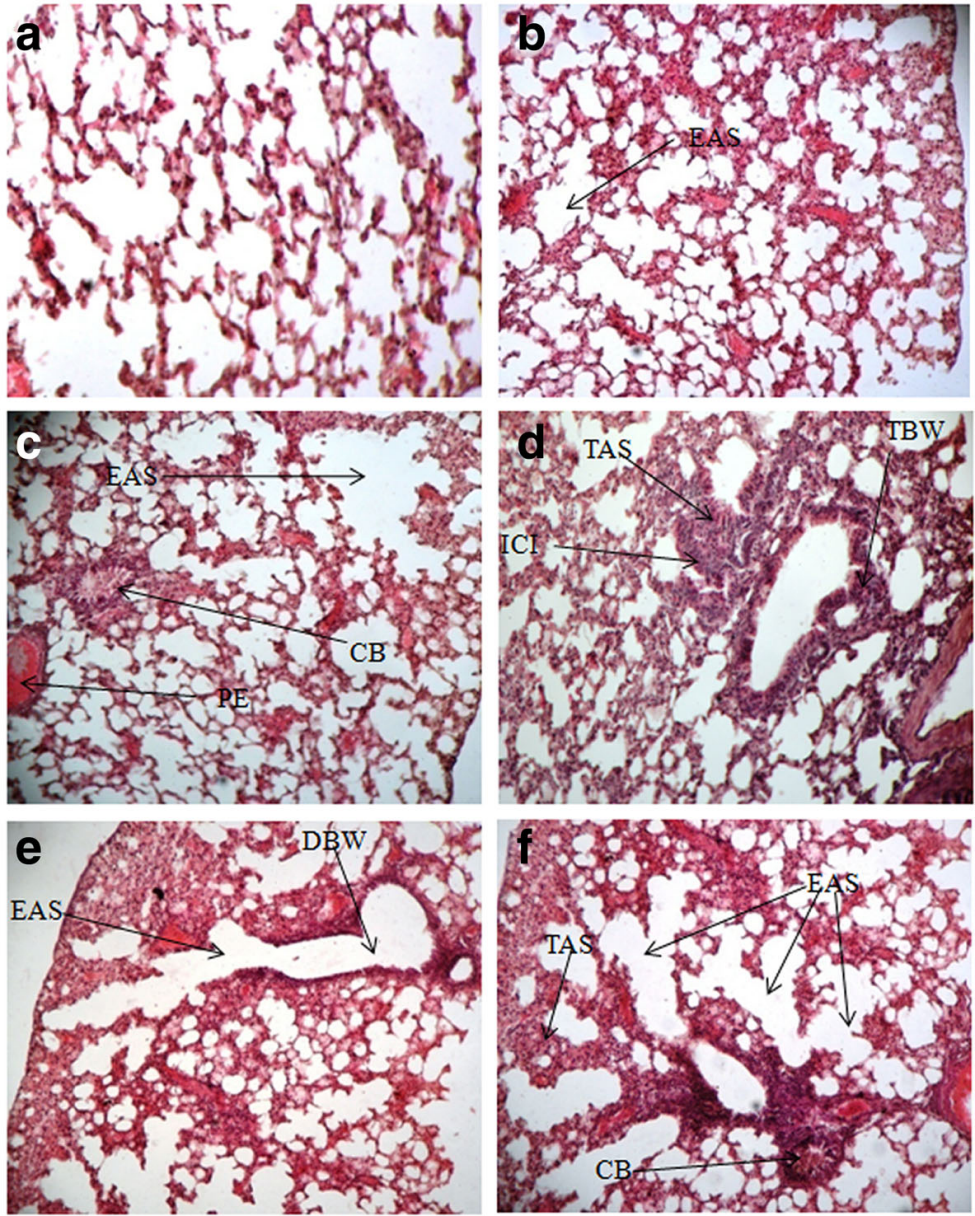
Histological section of lung tissue of mice inhaled with the mosquito coil smoke in the duration for 120 d/d. (**a**) Control mice; (**b**) ½ h inhalation/d; (**c**) 1 h inhalation/d; (**d**) 2 h inhalation/d and (**e**) & (**f**) 3 h inhalation/d. EAS indicates enlargement of air space, ICI indicates inflammatory cellular infiltration, CB represents constricted bronchi, PE represents pulmonary edema, TAS indicates thickening of alveolar septa, TBW indicates thickening of bronchiolar epithelial wall and DBW indicates disruption of bronchiolar epithelial wall. Sections were stained with hematoxylin and eosin (Original magnification, X400)

Pulmonary tissue of inhaled mice illustrates various degrees of histo-architectural changes in a doses dependent manner in comparison with that of control (Fig. 1). Transverse section of lung of control mice shows normal and compact organization with thin intra-alveolar septa and alveolar sacs (Fig. 1A). However, among of the smoke inhaled groups, mice, inhaled the MC smoke for 1/2 h/d show normal structure only with mild enlargement of air space (Fig. 1B). On the other hand, exposure of the smoke for 3 h/d results in mass infiltration of inflammatory cells (ICI) around the air way and in alveolar septa along with massive enlargement of air space (EAS Fig. 1E-F) by the disruption of inters alveolar septa and bronchiolar epithelial wall (DBW in Fig. 1E). The ruptured alveoli further join with each other and form large air space especially in the peripheral region of lung (Fig. 1E and F). It implies that air ventilation through the bronchi and bronchiole in treated mice might be considerably reduced due to constricted bronchi (CB in Fig. 1C and F). Besides these, marked form of hyperplasia was also observed by thickening of bronchiolar epithelial wall (TBW) as well as alveolar septa (TAS) in animal inhaled the smoke for 2 h to 3 h/d (Fig. 1D-F). Higher duration inhalation also resulted in pulmonary edema (PE in Fig. 1C-F).

### Histological changes in hepatic tissue

**Fig. 2:**
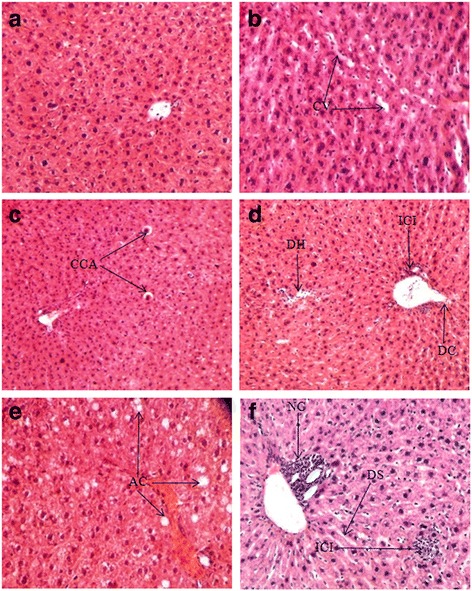
Histological section of live tissue of mice inhaled with the mosquito coil smoke in the duration for 120 d/d. (**a**) Control mice; (**b**) ½ h inhalation/d; (**c**) 1 h inhalation/d; (**d**) 2 h inhalation/d and (**e**) & (**f**) 3 h inhalation/d. CV indicates cytoplasmic vacuolation, CCA indicates condensation of cytoplasm of ongoing apoptotic cell, DH represents degeneration hepatocytes, ICI represents inflammatory cellular infiltration, DC indicates dilation of central vein, AC indicates apoptotic cells, NC indicates necrosis and DS is for dilation of sinusoid. Sections were stained with hematoxylin and eosin (Original magnification, X400)

Toxicological effects of MC smoke on hepatic tissue in control and smoke inhaled mice are shown in Fig. 2. Microscopic observation of control liver shows regular and compact configuration with well-organized hepatocytes and central vein (Fig. 2A). While, necrotic (NC) and apoptotic (AC) degeneration of hepatocytes along with infiltration of inflammatory cells (ICI) around the central vein and in the sinusoidal space were found in the liver of mice, exposed to the smoke of MC for 3 h and 2 h/day (fig. 2 D-F). Treated mice with higher duration of inhalation show degenerated hepatocytes (DH), dilation of central vein (DC) and dilation of sinusoid (DS) (Fig. 2D and F). Apoptotic characteristic such as condensation of cytoplasm (CCA) was observed in 1 h inhaled group (Fig. 2C). No significant histological changes but cytoplasmic vacuolation (CV) were found in mice exposed to smoke of MC for 1/2 h (Fig. 2B).

## Discussion

MC contains pyrethrin based insecticide but still widely being used in the tropical and subtropical region including Bangladesh as a common approach to protect mosquito from entering into home. MC generally has higher acceptance over liquid vaporizer due to its lower cost and ease of use. Upon burning, MC releases over 60 organic compounds, fine and ultrafine particles and heavy metals [5]. The smoke is composed of two phases: gas and particle phase [26]. During overnight burning, the smoke of particle phase containing carbon particles, heavy metals, aldehydes ultimately reaches peripheral region of lung and leading to progressive cellular injury and destruction of mucus membrane [9].

The present study demonstrates that critical biochemical parameters of blood were altered significantly in MC smoke inhaled animals. The activity of transaminases (ALT and AST) was increased significantly (at *P* < 0.05) in smoke inhaled animals (Table 1). Elevated activity of these hepatic enzymes is strongly correlated with hepatic injury [11]. It is either due to the direct effects of allethrin on the membrane phospholipids of hepatocytes and/or indirect effects caused due to the by-products derived from pyrethroid metabolism. Allethrin is membrane active substance, which acts upon membrane phospholipids and increases membrane fluidity [13] and leading to leak out cellular enzymes in extracellular matrix eventually increasing transaminases activity in blood. Elevated transaminase level has also been reported previously by Karthikeyan et al., (2006) in mice, exposed to mosquito repellent mat vapor [27].

The exposure of MC smoke resulted dose dependent depletion of BUN and total protein activity in all inhaled animals. This finding is also supported by a number of observations which demonstrated that kidney functions were not negatively altered in MC smoke inhaled rat [5, 11]. Similarly, the MC smoke has previously been reported to reduce serum total protein activity both in rodent animal and human subject [27, 28]. Higher protein degradation with subsequent reduction of total protein activity of blood in smoke inhaled subject has also been confirmed by marked increase of plasma free amino acid levels [28].

The elevated activity of some parameters of lipid profile in smoke inhaled mice in current study (Table 1) is not accord with the findings conducted by Narendra et al.,(2008), which demonstrates decreased plasma CHOL and LDL activity in human subjects, who inhaled the smoke of allethrine based MC regularly for long period of time [28]. Elevated LDL activity is considered to be the risk factor for cardiac condition, because it transports cholesterol from the liver to peripheral tissues. However, it is the first study that documented the raised cholesterol level in rodent animal treated with allethin based MC smoke via inhalation.

The oxidative damage, induced by ROS is the hallmark of cellular stress which modulates a number of signaling pathways including carcinogenesis, apoptosis, necrosis and inflammatory pathway. Free radical and volatile organic compounds present in the smoke of MC is considered as the potent contributor of oxidative DNA damage and tissue injury. Under these stress conditions, the histological organization of pulmonary and hepatic tissues becomes affected which led to severe organ injury. Histological study of lung tissue demonstrates extensive destruction of the alveolar septa and bronchial epithelial wall resulting in loss of alveoli as well as capillary surface area. Thus, the current study shows the clear evidence of severe emphysema as a result of long time inhalation of allethrin based MC smoke (Fig. 1E-F). The key event of the development of emphysema is the imbalance between proteases and anti-proteases, which is well established [29]. The ROS, induced by MC smoke, interferes with the antioxidant defense system [16], which would triggers inflammatory response in damage sites [30]. Furthermore, over expression of p53 in MC induced animals [16] is also considered as the potent contributor of pulmonary and hepatic inflammation. Under stress condition, p53 has been reported to activate pro-inflammatory cytokines at the site of stress induced pulmonary tissue in cigarette smoke inhaled rat [31]. However, the recruited inflammatory cells along with proteolytic enzymes act upon the anti-proteases (collagen and elastin) in alveolar region and inactivate them which in turn results in the destruction of alveolar septa and eventually develop emphysema [29]. Additional evidence has also confirmed the involvement of neutrophil in the degradation of elastin fibers with subsequent development of emphysema [32].

Emphysema in smoke inhaled animals is also accompanied by extensive thickening of alveolar septa and bronchial epithelial wall, which indicate hyperplasia (over tissue growth). Co-existence of emphysema and hyperplasia in pulmonary tissue was also observed in mice expressing TNF-α at elevated level [33]. Therefore, it can be postulated that current pulmonary injury would also due to the up-regulation of TNF-α in some extent. Because, elevated TNF-α level has been reported to deplete the total cellular glutathione (antioxidant enzyme) level in both in vitro and in vivo model [34, 35]. However, over tissue growth (TAS and TBW in Fig. 1D) in the current findings would also be due to extensive proliferation and deposition of two primary connective tissue components i,e. elastin and collagen in emphysematous areas [36]. Accumulation of inflammatory cells probably induces deposition of extracellular matrix in the sub epithelium region and contributes in thickening of bronchial epithelial wall [37]. The structure of epithelial lining of bronchi is converted from pseudo-stratified columnar ciliated epithelium into thickened constricted bronchi (CB in fig-1C and 1B), which might limit air passing through the bronchi to the peripheral region of lung- major clinical feature in patient with COPD [38]. The activation of fibroblasts by inflammatory cells and with subsequent thickening of bronchial epithelial wall and alveolar septa are considered as airway resistance and airway remodeling against ongoing destruction of alveolar septa [37].

The co-existence of necrotic and apoptotic mediated liver damage is the novel finding of this study (AC and NC in Fig. 2E-F). Necrotic characteristics were mainly found in higher duration inhaling groups (3 h and 2 h), probably due to allethrin induced toxicity on cell membranes. Necrotic cells death characteristically affects large fields of tissue (NC in figure- 2F) rather than a single cell and induce to release cytoplasmic contents into the surrounding cellular environment which leads to trigger inflammatory cells to be accumulated (NC in Fig. 2E-F).

Histological observation of hepatic tissue also reveals degeneration of hepatocytes in such a way, affecting single cell or small area of tissue without recruiting inflammatory cells: the critical feature of apoptotic cells (AC in Fig. 2E) [39]. Some hepatocytes of 1 h inhaled mice shows condensation of cytoplasm and chromatin- the key event of apoptosis mediated cell death (CCA in fig-2C). However, it is the first time study that we have observed apoptotic mediated cell death induced by the smoke of allethrin based MC. Oxidative DNA damage with subsequent up-regulation of p53 is the perquisite of mitochondria mediated apoptosis [40]. Several studies earlier reported that ROS induced DNA damage and up-regulation of p53 in MC smoke inhaled rat [15, 16]. Although, the mechanism and cellular features of apoptosis and necrosis are different, but both the phenomenon share a common a biochemical network described as “apoptosis-necrosis continuum” [41]. The interplay between apoptosis and necrosis may take place at the same time in the same tissue depending upon the types and degrees of death stimuli [42], which would also be supportive to the current dose dependent co-existence of apoptosis and necrosis in smoke inhaled tissues.

This study has some limitations relating to the conclusion of the results. Here, we stained the respiratory and hepatic tissue only with hematoxiline-eosin, though other staining technique such as DAPI staining could be done to observe apoptotic cells more precisely. In this study, we could not check the function of respiratory system such as X-ray analysis respiratory system due to lack of proper facilities. In spite of some confines, these findings would help to select proper medication for diseases induced by the mosquito coil smoke, and this study gives the proposal to modify the chemical structure of the insecticide for avoiding unexpected effects on human body.

## Conclusion

In this study, we found the biochemical and histological effects of the mosquito coil smoke using mice model. Biochemically, inhalation of the mosquito coil smoke altered dose dependently in all of the blood parameters tested. In histological approach, the progressive destruction of lung tissue and sequential necrotic degeneration of liver tissue were newly characterized as toxicological phenomena of the mosquito coil smoke.

## Abbreviations

ALT: Alanine transaminase
AST: Aspartate transaminase
BUN: Blood urea nitrogen
CHOL: Cholesterol
COPD: Chronic obstructive pulmonary diseases
DAPI: 4′,6-diamidino-2-phenylindole
dl: deciliter
g: gram
HDL: High density lipoprotein
L: Liter
LDL: Low density lipoproteins
MC: Mosquito coil
ml: milliliter
NC: Necrosis
PARP: Poly (ADP ribose) polymerase
ROS: Reactive oxygen
rpm: rotation per minutes; species
TG: Triglycerides
TNF: Tumor necrosis factor
U: Unit
